# Clinical Outcomes of Respiratory Syncytial Virus Versus Influenza Pneumonia in Critically Ill Adults

**DOI:** 10.3390/life16091533

**Published:** 2026-09-15

**Authors:** Josef Yayan

**Affiliations:** Department of Internal Medicine, Division of Pulmonary, Allergy, and Sleep Medicine, Helios University Hospital Wuppertal, Witten/Herdecke University, Heusnerstr. 40, 42283 Wuppertal, Germany; josef.yayan@hotmail.com

**Keywords:** respiratory syncytial virus, influenza, viral pneumonia, intensive care unit, epidemiology, in-hospital mortality, prognosis, respiratory infections

## Abstract

Background: Respiratory syncytial virus (RSV) and influenza can both cause severe viral pneumonia requiring intensive care. Influenza is supported by established vaccination programs and antiviral therapy, whereas outcomes of RSV infection among critically ill adults remain less well characterized. This study compared clinical presentation, treatment, and outcomes between ICU admissions classified as RSV pneumonia and those classified as influenza pneumonia. Methods: A retrospective cohort was assembled from MIMIC-IV data covering 2008–2019. The analytical cohort comprised 201 infection-specific ICU observations classified as RSV pneumonia (*n* = 40) or influenza pneumonia (*n* = 161). Demographic characteristics, clinical measurements, treatments, and hospital outcomes were compared. Multivariable logistic regression was used to examine the association between infection type and in-hospital mortality, with adjustment for age, sex, and serum lactate. Internal model validation used 300 bootstrap resamples. Results: The RSV group was older than the influenza group (66.7 vs. 59.1 years; *p* = 0.009), had a longer ICU stay (median 3.0 [IQR 2.0–7.0] vs. 2.0 [IQR 1.0–4.0] days; *p* = 0.003), and had a higher admission heart rate (*p* = 0.009). Oseltamivir was recorded in 89.4% of influenza observations and 2.5% of RSV observations (*p* < 0.001). In-hospital mortality was 30.0% in the RSV group and 11.8% in the influenza group (*p* = 0.0091). In the primary adjusted model, RSV pneumonia was associated with higher odds of in-hospital death (adjusted OR 6.03, 95% CI 2.08–17.49; *p* = 0.001), as was increasing serum lactate (adjusted OR 1.46, 95% CI 1.24–1.74; *p* < 0.001). The apparent AUC was 0.864, and the optimism-corrected AUC after bootstrap validation was 0.845. Sensitivity analyses indicated that the magnitude of the RSV association varied with covariate adjustment. Conclusions: RSV pneumonia was associated with higher in-hospital mortality and a longer ICU stay than influenza pneumonia in this ICU cohort. The mortality association remained after adjustment for the variables included in the primary model, although its magnitude was sensitive to model specification. Given the observational design, these findings should not be interpreted as causal. They underscore the clinical burden of severe RSV infection in adults and support further investigation in larger cohorts with standardized virological testing and measures of acute illness severity.

## 1. Introduction

Severe pneumonia caused by respiratory viruses remains an important reason for admission to an intensive care unit (ICU), particularly in older adults and in people with substantial comorbidity [1]. Influenza has long been recognized as a major viral cause and can be addressed through established vaccination programs and specific antiviral treatment [2]. Respiratory syncytial virus (RSV), historically viewed mainly as a childhood pathogen, is now also recognized as an important cause of serious lower respiratory tract disease in adults, especially among older and critically ill patients [3,4].

Adult RSV infection accounts for a substantial burden of hospitalization and intensive care use. In selected high-risk populations, reported mortality has been similar to, or higher than, that associated with influenza [5,6]. Even so, the clinical course and prognosis of RSV among critically ill adults remain less well characterized than those of influenza. Compared with influenza, RSV testing has historically been less routine, adult surveillance data are more limited, and pathogen-specific therapeutic options remain restricted [7].

Clinical outcomes may differ between the two infections because of differences in host characteristics, viral disease patterns, and the availability of pathogen-specific treatment. Although direct comparisons between RSV and influenza in hospitalized adults have become more available, evidence specifically focused on critically ill populations remains limited [7,8]. Differences in case mix, diagnostic testing, and clinical management across studies further complicate direct comparison of outcomes.

The present analysis therefore compared ICU observations classified as RSV pneumonia or influenza pneumonia in the Medical Information Mart for Intensive Care IV (MIMIC-IV) database. The groups were compared with respect to demographic characteristics, initial clinical findings, treatment patterns, and hospital outcomes. Multivariable logistic regression with bootstrap internal validation was additionally used to examine the association between infection type and in-hospital mortality.

## 2. Materials and Methods

### 2.1. Study Design and Data Source

Data were obtained from version 3.1 of the Medical Information Mart for Intensive Care IV (MIMIC-IV), a de-identified clinical database from Beth Israel Deaconess Medical Center in Boston, MA, USA. The study period covered admissions from 2008 through 2019 [9]. MIMIC-IV provides linked information on patient demographics, bedside observations, laboratory testing, medications, microbiology, and clinical outcomes. Access to the database required completion of the Collaborative Institutional Training Initiative (CITI) training and fulfillment of the applicable PhysioNet credentialing requirements. Because the data are de-identified, individual informed consent was not required.

### 2.2. Study Population

Eligible observations were ICU admissions of adults aged 18 years or older classified as RSV pneumonia or influenza pneumonia. Case ascertainment was based primarily on ICD-9/ICD-10 diagnostic coding, with available microbiological information used as supporting evidence. The ICD-10 codes represented in the analytical cohort were J12.1 and B97.4 for RSV and J09.X1, J09.X2, J10.08, J10.1, J10.81, J11.08, and J11.1 for influenza. Microbiological information was not uniformly available and was therefore not required for inclusion. Consequently, microbiological confirmation, the specific diagnostic assay used, and the timing of testing relative to ICU admission could not be determined consistently across the entire cohort. One ICU admission met the classification criteria for both RSV and influenza pneumonia and was therefore represented once in each infection group. The primary analysis retained both infection-specific observations. A sensitivity analysis excluded both observations corresponding to this overlapping ICU admission.

### 2.3. Data Collection

For each included ICU admission, the analysis captured the following information:

- age and sex as demographic variables;
- the earliest available ICU values for heart rate, respiratory rate, temperature, and peripheral oxygen saturation (SpO_2_);
- available laboratory results, including leukocyte count, albumin, serum lactate, and C-reactive protein;
- receipt of oseltamivir, azithromycin, ceftriaxone, and invasive mechanical ventilation.

The earliest available bedside measurements after ICU admission were used for the vital-sign analyses. Values were screened for physiological plausibility before statistical testing. The accepted ranges were 20–250 beats/min for heart rate, 5–80 breaths/min for respiratory rate, 50–100% for SpO_2_, and 30–42 °C for temperature. Measurements outside these limits were omitted from the relevant analysis, resulting in exclusion of one heart-rate value, four respiratory-rate values, and one SpO_2_ value. Temperatures recorded in Fahrenheit were converted to degrees Celsius. Information on supplemental oxygen at the time of the recorded SpO_2_ measurement was not available in the analytical dataset.

Comorbidity burden was assessed using an ICD-based Charlson Comorbidity Index. ICD-coded immunocompromised status was additionally derived for sensitivity analysis. These variables were used to examine whether adjustment for underlying comorbidity materially changed the association between infection type and in-hospital mortality. Admission heart rate was used as an available marker of acute physiological severity in a separate sensitivity analysis.

In-hospital death was defined as the primary outcome. ICU length of stay and use of invasive mechanical ventilation were secondary outcomes. Mechanical ventilation was retained as a descriptive treatment/outcome variable but was omitted from the mortality models because it may occur after ICU admission and adjustment for it could introduce overadjustment.

### 2.4. Statistical Analysis

Summary statistics were chosen according to variable type and distribution. Continuous measures are reported as mean ± standard deviation (SD), except for ICU length of stay and peripheral oxygen saturation (SpO_2_), which are presented as median and interquartile range (IQR) because of their distributional characteristics. Categorical data are shown as counts and percentages. RSV and influenza groups were compared using Student’s *t*-test or the Mann–Whitney *U* test for continuous variables and the chi-square test or Fisher’s exact test for categorical variables, as appropriate. Comparisons were considered exploratory; consequently, no multiplicity correction was applied, and *p*-values were interpreted together with standardized mean differences. Descriptive analyses used the observations available for each variable without imputation. The multivariable mortality analyses used complete cases for the covariates included in each respective model. The BigQuery SQL used for cohort construction and variable derivation, the Python code used for data cleaning and statistical analyses, and the corresponding Python package versions are provided in the Supplementary Materials (Supplementary Codes S1 and S2 and the accompanying requirements file).

To quantify the association between infection type and in-hospital death, a multivariable logistic regression model was fitted with RSV versus influenza, age, sex, and serum lactate as predictors. These covariates were selected on the basis of clinical relevance and availability in the analytical dataset. Effect estimates are reported as adjusted odds ratios (ORs) with 95% confidence intervals (CIs). Additional models were fitted sequentially, beginning with the unadjusted association and then adding age and sex before inclusion of serum lactate. A separate analysis tested the interaction between age and infection type. Because the number of deaths was limited, Firth penalized logistic regression was used to assess the robustness of the principal estimates to sparse-data bias.

Additional sensitivity analyses examined the influence of comorbidity burden and acute physiological severity on the estimated association between RSV pneumonia and in-hospital mortality. Models additionally incorporated the Charlson Comorbidity Index and ICD-based immunocompromised status. In a separate complete-case sensitivity model, admission heart rate was included together with age, sex, and serum lactate as an available marker of acute physiological severity. A further sensitivity analysis excluded ICU admission with overlapping RSV and influenza classification.

Discrimination of the final logistic model was summarized by the area under the receiver operating characteristic (ROC) curve (AUC). McFadden’s pseudo-R^2^ and the likelihood-ratio test were used as additional measures of overall model performance. Internal validation relied on 300 bootstrap samples generated with a fixed random seed of 42. No prospective sample-size or power calculation was performed because this retrospective study included the fixed set of eligible observations available in the MIMIC-IV cohort during the predefined study period. All tests were two-sided, with *p* < 0.05 used as the threshold for statistical significance. VassarStats (Vassar College, Poughkeepsie, NY, USA) was used for descriptive and univariable analyses. Python version 3.13.5 (Python Software Foundation, Wilmington, DE, USA) was used for multivariable and sensitivity analyses. The Python environment included pandas version 2.2.3, NumPy version 2.3.5, SciPy version 1.17.0, statsmodels version 0.14.6, and scikit-learn version 1.8.0. Multivariable logistic regression, Firth penalized logistic regression, interaction testing, ROC analysis, and bootstrap internal validation were performed in Python.

## 3. Results

### 3.1. Patient Cohort

**Figure 1:**
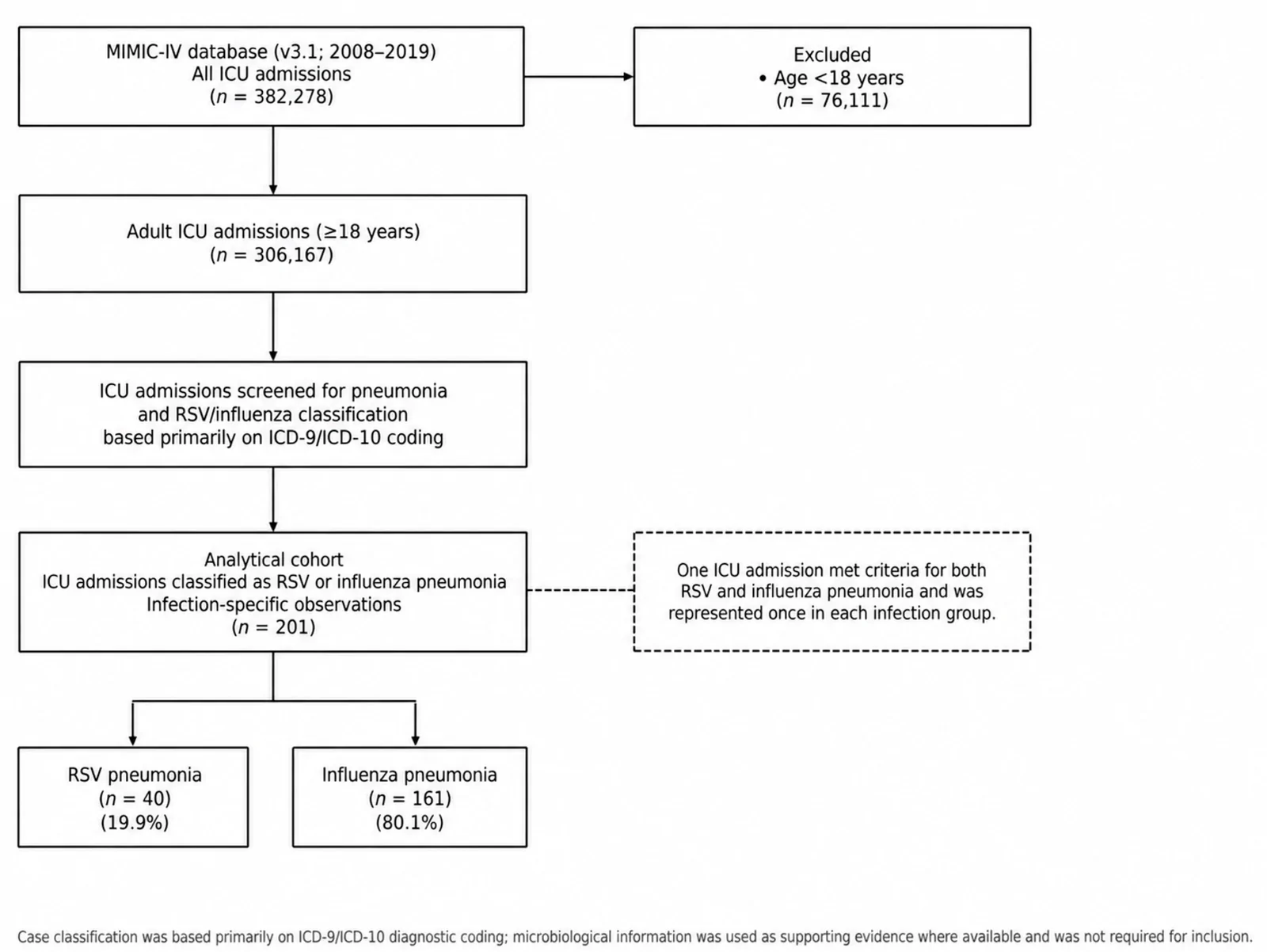
Patient-selection flow diagram. ICU admissions recorded in MIMIC-IV during 2008–2019 were screened according to the study eligibility criteria. Exclusions included individuals younger than 18 years, ICU admissions without pneumonia, and observations that did not meet the definition of RSV or influenza pneumonia based on diagnostic coding and available microbiological evidence. The analytical cohort comprised 201 infection-specific observations, including 40 classified as RSV pneumonia and 161 as influenza pneumonia. One ICU admission had overlapping RSV and influenza classification and was represented in both infection groups.

Application of the eligibility criteria to ICU admissions recorded in MIMIC-IV between 2008 and 2019 yielded 201 infection-specific observations classified as RSV or influenza pneumonia (Figure 1). Of these, 40 observations (19.9%) were classified as RSV pneumonia and 161 (80.1%) as influenza pneumonia. One ICU admission had overlapping RSV and influenza classification and was therefore represented in both infection groups.

### 3.2. Baseline Characteristics

Table 1 summarizes demographic data, clinical measurements, laboratory findings, outcomes, and selected treatments. Mean age was higher in the RSV group than in the influenza group (66.7 ± 15.6 vs. 59.1 ± 17.6 years; *p* = 0.009). Admission heart rate was likewise higher with RSV (132.9 ± 35.2 vs. 117.0 ± 23.6 beats/min; *p* = 0.009). Oxygen saturation showed a pronounced ceiling effect. SpO_2_ was exactly 100% in 37 of 40 RSV observations (92.5%) and 129 of 160 influenza observations (80.6%), with a median of 100% (IQR, 100–100%) in both groups. Only one observation in the influenza group and none in the RSV group had an SpO_2_ < 95%, precluding a meaningful categorical comparison at this threshold. Information on supplemental oxygen at the time of SpO_2_ measurement was not available in the analytical dataset. Respiratory rate, temperature, albumin, lactate, and C-reactive protein did not differ significantly between groups. Leukocyte count also did not differ significantly, although this comparison was based on only 32 available observations (RSV, *n* = 7; influenza, *n* = 25) and should therefore be interpreted cautiously. ICU stay was longer in the RSV group, with a median of 3.0 days (IQR, 2.0–7.0) compared with 2.0 days (IQR, 1.0–4.0) in the influenza group (*p* = 0.003).

Missing data were limited for vital signs but more substantial for selected laboratory variables. Heart rate was missing in 1 observation (0.5%), respiratory rate in 4 (2.0%), and SpO_2_ in 1 (0.5%). Leukocyte count was missing in 169 observations (84.1%; RSV: 33/40 [82.5%]; influenza: 136/161 [84.5%]), albumin in 67 (33.3%; RSV: 11/40 [27.5%]; influenza: 56/161 [34.8%]), and serum lactate in 40 (19.9%; RSV: 9/40 [22.5%]; influenza: 31/161 [19.3%]). C-reactive protein data were complete. Consequently, the primary multivariable mortality model including lactate was based on 161 complete observations and 30 in-hospital deaths. The single death excluded from the complete-case model because of missing lactate occurred in the RSV group.

**Figure 2:**
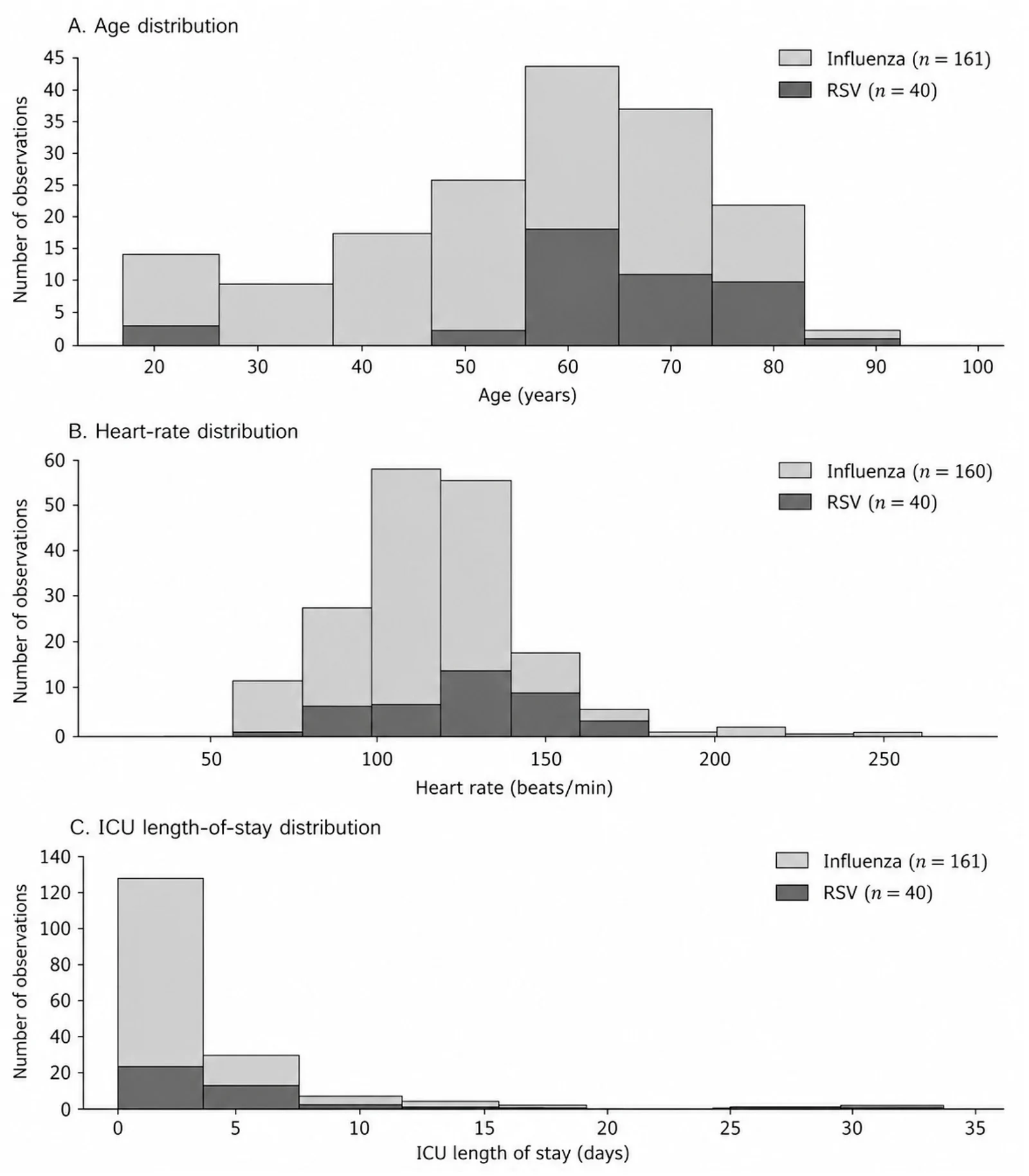
Distribution of age, heart rate at ICU admission, and ICU length of stay according to infection group. Heart-rate data were available for 160 influenza and 40 RSV observations. Histograms show the distributions for influenza and RSV pneumonia observations.

Figure 2 displays the group distributions for age, ICU-admission heart rate, and ICU length of stay.

### 3.3. Treatment Patterns and Interventions

Use of several treatments differed between the infection groups (Table 1). Oseltamivir was recorded in 89.4% of influenza observations compared with 2.5% of RSV observations (*p* < 0.001). In contrast, azithromycin was recorded more frequently in the RSV group than in the influenza group (70.0% vs. 31.7%; *p* < 0.001). Ceftriaxone use was similar between groups (40.0% vs. 32.9%; *p* = 0.458), as was the use of invasive mechanical ventilation (42.5% vs. 32.9%; *p* = 0.273).

### 3.4. Clinical Outcomes

**Figure 3:**
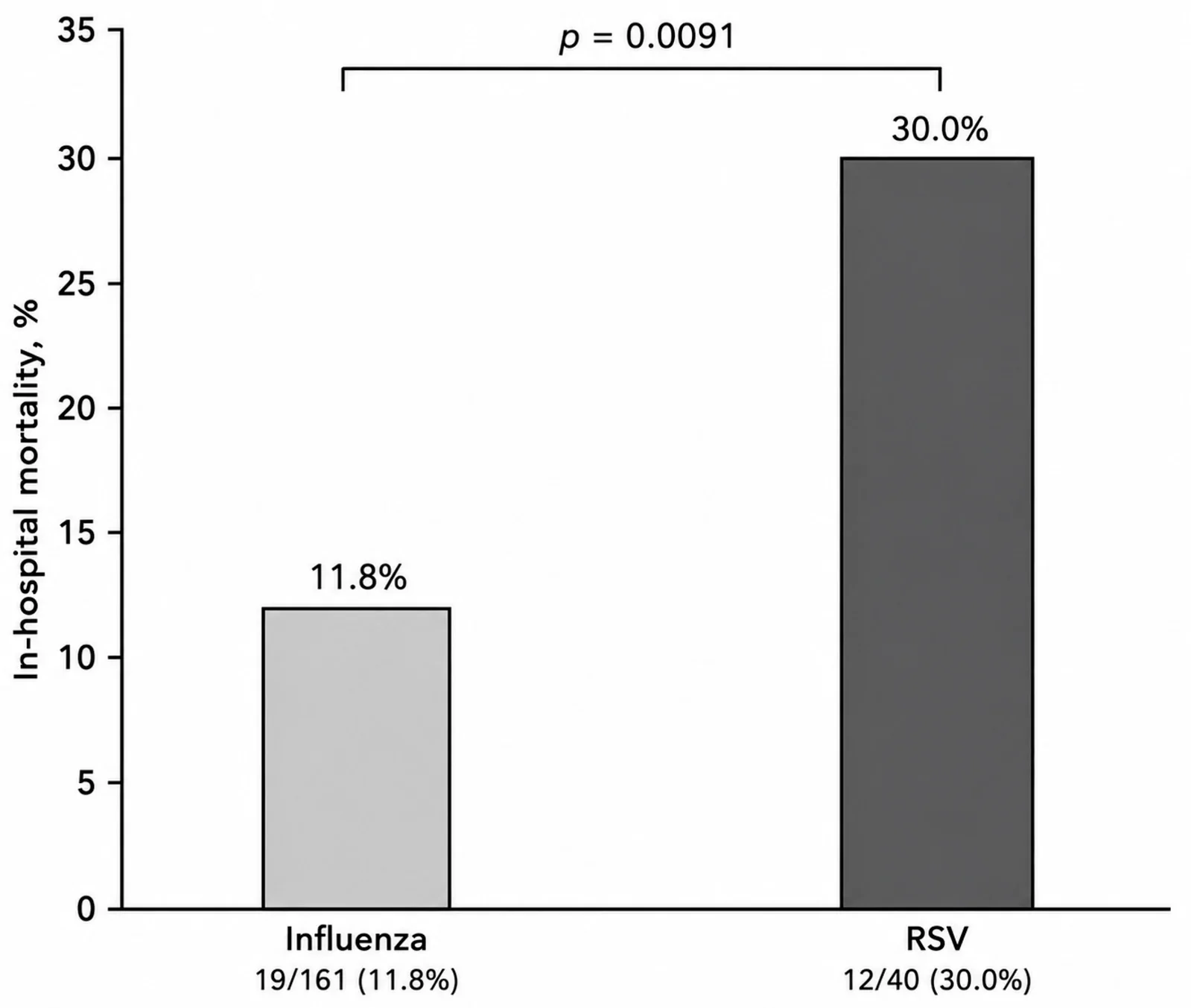
In-hospital mortality according to viral pneumonia type. In-hospital mortality was 30.0% (12/40) in the RSV group and 11.8% (19/161) in the influenza group (*p* = 0.0091). The *p*-value represents the unadjusted between-group comparison.

Hospital outcomes are also reported in Table 1. In-hospital death occurred in 12 of 40 RSV observations (30.0%) and 19 of 161 influenza observations (11.8%; *p* = 0.0091) (Figure 3). Median ICU length of stay was 3.0 days (IQR, 2.0–7.0) in the RSV group compared with 2.0 days (IQR, 1.0–4.0) in the influenza group (*p* = 0.003).

### 3.5. Multivariable Logistic Regression

**Figure 4:**
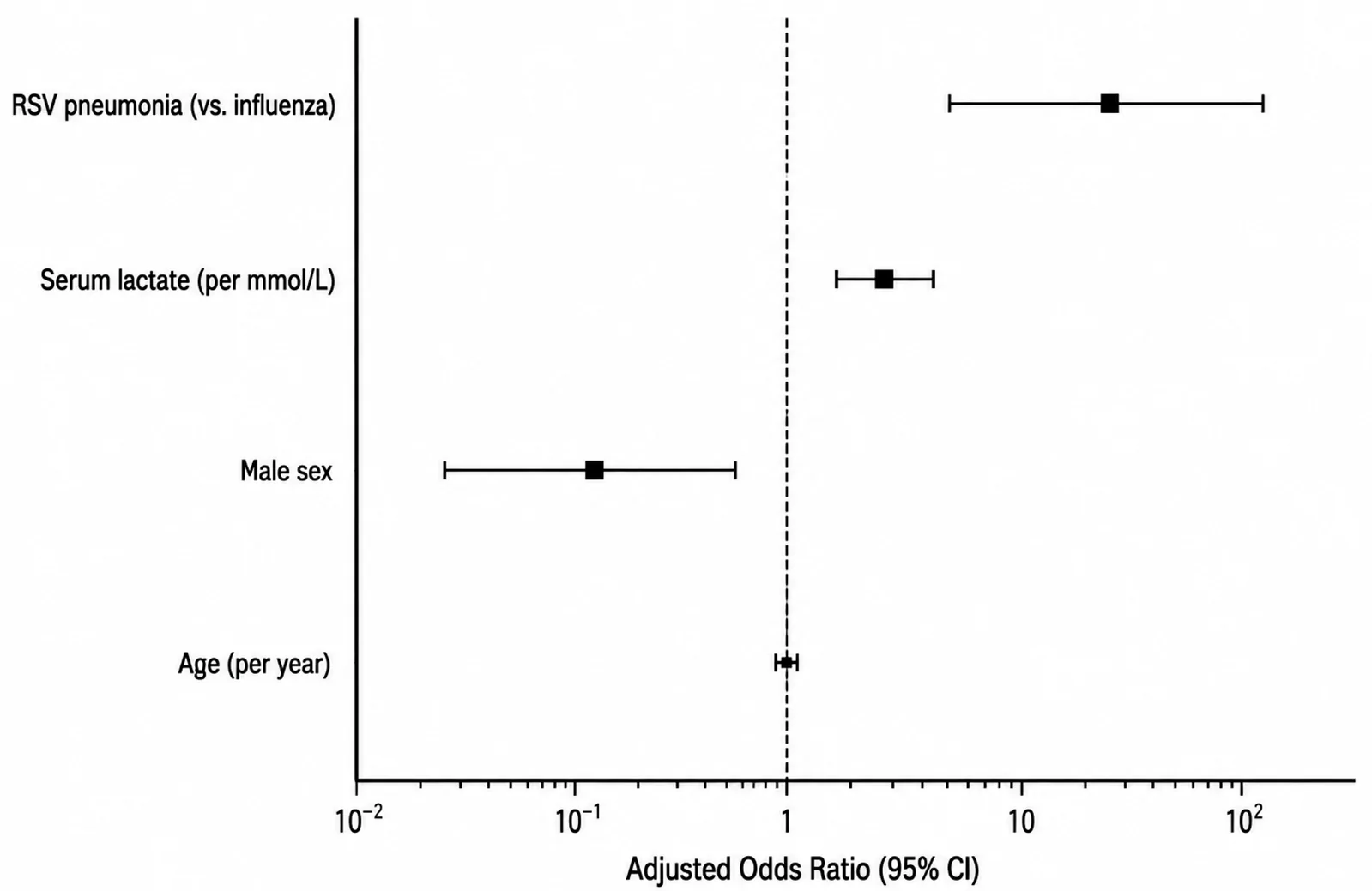
Forest plot of adjusted odds ratios (ORs) for in-hospital mortality from the final multivariable logistic regression model. Squares represent adjusted ORs and horizontal lines indicate 95% confidence intervals (CIs). The vertical dashed line at OR = 1 indicates the null value. Influenza pneumonia and female sex served as the reference categories. The model was based on 161 complete observations with 30 in-hospital deaths.

Table 2 presents the final multivariable estimates, which are also visualized in Figure 4. Complete information on infection type, age, sex, and serum lactate was available for 161 observations, among which 30 in-hospital deaths occurred. After adjustment for age, sex, and serum lactate, RSV pneumonia was associated with higher odds of in-hospital death than influenza pneumonia (adjusted OR 6.03, 95% CI 2.08–17.49; *p* = 0.001). Each 1 mmol/L increase in serum lactate was also associated with higher odds of in-hospital death (adjusted OR 1.46, 95% CI 1.24–1.74; *p* < 0.001). Male sex was associated with lower odds of in-hospital death (adjusted OR 0.24, 95% CI 0.08–0.70; *p* = 0.009), whereas age was not independently associated with the outcome (*p* = 0.861).

The age-by-infection interaction was not statistically significant (OR 0.96, 95% CI 0.89–1.03; *p* = 0.225). In the sequential models, the unadjusted OR for RSV pneumonia was 3.20 (95% CI 1.40–7.34; *p* = 0.006), remained similar after adjustment for age and sex (OR 3.13, 95% CI 1.33–7.34; *p* = 0.009), and increased after serum lactate was added to the model (OR 6.03, 95% CI 2.08–17.49; *p* = 0.001) (Table 3). This change indicates that the magnitude of the RSV estimate was sensitive to covariate adjustment, particularly the inclusion of serum lactate. Firth penalized logistic regression yielded a comparable estimate for RSV pneumonia (OR 5.59, 95% CI 1.98–15.76), indicating that the direction of the association was not substantially altered by penalization.

### 3.6. Model Performance

**Figure 5:**
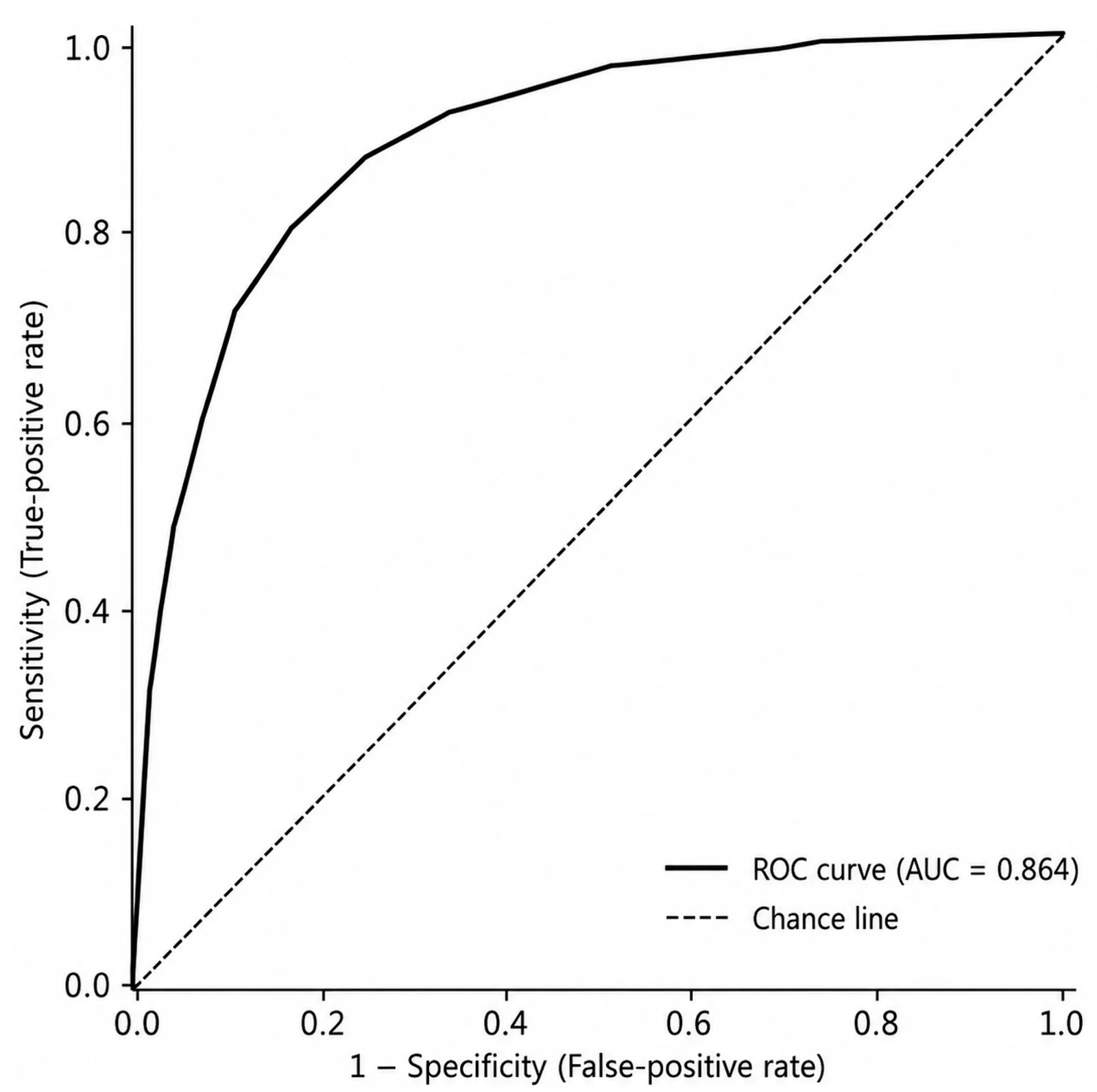
ROC curve for the final multivariable logistic regression model of in-hospital mortality among ICU observations classified as RSV or influenza pneumonia. The apparent AUC was 0.864; internal validation using 300 bootstrap resamples yielded an optimism-corrected AUC of 0.845.

Model performance is summarized in Table 4 and depicted by the ROC curve in Figure 5. The apparent AUC for discrimination of in-hospital mortality was 0.864. After internal validation using 300 bootstrap resamples, the optimism-corrected AUC was 0.845, indicating only modest optimism in the apparent discrimination.

In sensitivity analyses, the magnitude of the association between RSV pneumonia and in-hospital mortality varied according to covariate adjustment. The age- and sex-adjusted OR was 3.13 (95% CI, 1.33–7.34), whereas additional adjustment for serum lactate increased the estimate to 6.03 (95% CI, 2.08–17.49) (Table 3). When admission heart rate was additionally included as an available marker of acute physiological severity, the RSV estimate was attenuated to 3.67 (95% CI, 1.17–11.53) (Table 5). These findings indicate that the magnitude of the estimated association was sensitive to model specification and to adjustment for available markers of acute physiological severity.

In the Firth penalized logistic regression model, the corresponding ORs were 5.59 (95% CI, 1.98–15.76; *p* = 0.001) for RSV pneumonia, 1.002 (95% CI, 0.972–1.033; *p* = 0.896) for age, 0.267 (95% CI, 0.096–0.743; *p* = 0.011) for male sex, and 1.421 (95% CI, 1.209–1.670; *p* < 0.001) for serum lactate (Table 6).

Sensitivity analyses were performed to examine the influence of comorbidity burden and acute physiological severity on the estimated association. Adjustment for the Charlson Comorbidity Index resulted in an RSV OR of 3.10 (95% CI, 1.32–7.29; *p* = 0.009), similar to the age- and sex-adjusted estimate of 3.13 (95% CI, 1.33–7.34; *p* = 0.009). Additional adjustment for ICD-based immunocompromised status yielded a comparable estimate (OR, 3.05; 95% CI, 1.30–7.15; *p* = 0.010). In a separate complete-case sensitivity model that additionally included admission heart rate and serum lactate, the RSV estimate was attenuated but remained statistically significant (OR, 3.67; 95% CI, 1.17–11.53; *p* = 0.026) (Table 5).

Exclusion of the single ICU admission with overlapping RSV and influenza classification did not materially alter the association between RSV pneumonia and in-hospital mortality. In the corresponding complete-case model, the RSV OR was 6.34 (95% CI, 2.17–18.55; *p* < 0.001), based on 159 observations with 30 in-hospital deaths.

## 4. Discussion

In this ICU cohort, RSV pneumonia and influenza pneumonia showed meaningful differences in outcome. The RSV group had higher in-hospital mortality and a longer ICU stay than the influenza group. The association between RSV pneumonia and mortality persisted after adjustment for age, sex, and serum lactate, although its magnitude varied across model specifications. Together, these findings add to evidence that RSV can cause severe disease in adults requiring intensive care [7,10,11,12]. The expanding use of RSV vaccines in older adults may alter the future profile of severe RSV cases reaching the ICU [13,14,15,16,17].

The 30% in-hospital mortality observed in the RSV group lies toward the upper end of estimates previously reported for severe RSV among hospitalized or critically ill adults [7,10,11,12,18]. In this cohort, mortality was more than twice that observed in the influenza group. Other multicenter investigations have reported smaller differences or comparable mortality between RSV and influenza [7,12]. Differences in case mix, thresholds for ICU admission, illness severity, diagnostic practices, and local management may contribute to variation across studies. Although the association between RSV pneumonia and mortality remained after multivariable adjustment, the observational design precludes causal interpretation.

ICU length of stay was also longer in the RSV group. A prolonged ICU stay may reflect persistent respiratory dysfunction, slower recovery, or greater overall illness burden [10,11,12,18,19,20]. Nearly nine of ten influenza observations included oseltamivir use, whereas oseltamivir was recorded in only one RSV observation. This contrast is consistent with differences in available pathogen-specific treatment options [3,4]. RSV-directed antiviral strategies remain under investigation and are not established as routine treatment for critically ill adults [3,4]. ICU length of stay should therefore be interpreted primarily as an outcome reflecting illness burden and recovery rather than as a baseline determinant of prognosis.

Most routine clinical and laboratory measurements did not clearly distinguish RSV from influenza at ICU admission. Heart rate was higher in the RSV group, whereas respiratory rate, temperature, SpO_2_, albumin, lactate, and C-reactive protein showed no statistically significant between-group differences. Leukocyte count also did not differ significantly, although this comparison was based on only 32 available observations and should be interpreted cautiously. Serum lactate nevertheless emerged as an independent correlate of in-hospital mortality in the multivariable model, consistent with its established prognostic relevance in severe pneumonia [21]. These findings suggest that routinely available bedside and laboratory variables alone may have limited ability to distinguish the two infections, supporting the role of virological testing in establishing the diagnosis [3,7].

The change in the RSV effect estimate after adjustment for serum lactate warrants particular caution. Lactate concentrations were descriptively lower in the RSV group despite higher mortality, and inclusion of lactate increased the RSV OR from 3.13 to 6.03. Adjustment for overall comorbidity burden and ICD-based immunocompromised status had little influence on the estimate, whereas additional adjustment for admission heart rate attenuated the RSV OR to 3.67. Taken together, these sensitivity analyses indicate that the magnitude of the estimated association was sensitive to model specification, particularly to adjustment for available markers of acute physiological severity. The adjusted OR should therefore be interpreted as a conditional, model-dependent estimate rather than as a fixed measure of the effect of RSV infection, and residual confounding by acute illness severity cannot be excluded.

The final logistic model showed an apparent AUC of 0.864, which decreased modestly to 0.845 after correction for optimism using 300 bootstrap resamples. This internal validation suggests that discrimination was relatively stable within the analytical dataset, but it does not provide evidence of performance in other populations. External validation would be required before considering the model for broader clinical application.

The present findings extend real-world evidence on severe RSV pneumonia in adults requiring intensive care [7,10,11,12,18,19,20]. Compared with influenza pneumonia, RSV pneumonia was associated with higher in-hospital mortality and a longer ICU stay in this cohort. However, the magnitude of the mortality association varied with covariate adjustment, underscoring the need for cautious interpretation and validation in larger cohorts with more comprehensive measures of acute illness severity. These findings support continued attention to prevention, accurate viral diagnosis, and improved therapeutic strategies for severe RSV disease in adults [3,4,13,14,15,16,17].

## 5. Limitations

Several limitations should be considered when interpreting these findings. First, the retrospective observational design is susceptible to selection bias and residual confounding that cannot be fully addressed through statistical adjustment. This is particularly relevant because the magnitude of the association between RSV pneumonia and in-hospital mortality varied across model specifications. Adjustment for serum lactate increased the RSV effect estimate, whereas additional adjustment for admission heart rate attenuated it. Although adjustment for comorbidity burden and ICD-based immunocompromised status had little influence on the estimate, residual confounding by acute illness severity cannot be excluded.

Second, all data originated from a single tertiary-care institution. Patient characteristics, diagnostic practices, thresholds for ICU admission, and treatment patterns may differ across institutions, limiting the generalizability of the findings to other hospitals and healthcare systems.

Third, the RSV group was relatively small, with only 40 observations. The resulting estimates were therefore imprecise, as reflected by the wide confidence intervals, and the study may have had limited ability to detect more modest between-group differences in baseline characteristics and secondary outcomes.

Fourth, case ascertainment relied on ICD-coded diagnoses together with microbiological information where available. Virological confirmation was not uniformly available, and misclassification of infection status therefore cannot be excluded. Differences in the frequency or timing of virological testing for RSV and influenza may also have resulted in differential case ascertainment between the two infection groups.

Fifth, comprehensive measures of acute illness severity, such as the Sequential Organ Failure Assessment (SOFA) and Acute Physiology and Chronic Health Evaluation (APACHE) scores, were not consistently available for the analytical cohort and could not be incorporated into the adjusted models. Admission heart rate and serum lactate captured only selected aspects of acute physiological severity and cannot substitute for validated multidimensional severity scores.

Sixth, missingness was substantial for several laboratory variables, particularly leukocyte count, albumin, and serum lactate. Descriptive analyses were based on available observations, whereas regression models requiring lactate were restricted to complete cases. This may have introduced additional selection and reduced precision. Information on supplemental oxygen at the time of SpO_2_ measurement was also unavailable, limiting interpretation of the pronounced ceiling effect observed for oxygen saturation.

Finally, treatment exposure was not protocolized; antiviral and antibacterial prescribing reflected routine clinical decisions and institutional practice. The observed treatment differences therefore should not be interpreted as evidence of treatment effects. Bootstrap resampling provided internal validation of model discrimination but does not establish transportability to other populations. Independent external validation would be required before considering the mortality model for broader clinical application.

## 6. Conclusions

Among ICU observations classified as RSV or influenza pneumonia, RSV pneumonia was associated with higher in-hospital mortality and a longer ICU stay. The association with mortality remained after adjustment for age, sex, and serum lactate, although its magnitude varied across model specifications. These observational findings should not be interpreted as causal but underscore the clinical burden of severe RSV infection in adults. Accurate virological diagnosis, appropriate supportive management, and continued development of RSV-directed therapies remain important. Confirmation in larger prospective multicenter cohorts incorporating standardized viral testing, validated measures of acute illness severity, and contemporary treatment data are needed to better define the prognosis and clinical implications of severe RSV pneumonia in adults.

## Figures and Tables

**Table 1:** Demographic, clinical, laboratory, outcome, and treatment characteristics of ICU observations classified as RSV pneumonia or influenza pneumonia. Continuous variables are reported as mean ± SD, except ICU length of stay and peripheral oxygen saturation (SpO_2_), which are presented as median (IQR); categorical variables are given as *n* (%). Group comparisons used Student’s *t*-test or the Mann–Whitney *U* test for continuous variables and the chi-square test or Fisher’s exact test for categorical variables, as appropriate. Standardized mean differences (SMDs) describe the magnitude of between-group differences.

Variable	Influenza (*n* = 161)	RSV (*n* = 40)	SMD	*p*-Value
Demographics				
Age, years	59.1 ± 17.6	66.7 ± 15.6	0.44	0.009
Female sex, *n* (%)	84 (52.2)	22 (55.0)	0.06	0.860
Clinical characteristics				
ICU length of stay, days	2.0 (1.0–4.0)	3.0 (2.0–7.0)	0.76	0.003
Heart rate, beats/min	117.0 ± 23.6	132.9 ± 35.2	0.60	0.009
Respiratory rate, breaths/min	33.89 ± 8.9	35.59 ± 6.51	0.20	0.180
Temperature, °C	37.72 ± 0.83	37.97 ± 0.98	0.29	0.139
Oxygen saturation, %, median (IQR)	100 (100–100)	100 (100–100)	0.23	0.077
Leukocyte count, ×10^9^/L	10.83 ± 0.66	10.34 ± 0.49	−0.77	0.053
Albumin, g/dL	3.32 ± 0.57	3.41 ± 0.66	0.14	0.530
Lactate, mmol/L	3.27 ± 3.84	2.58 ± 1.90	−0.19	0.685
C-reactive protein, mg/dL	2.67 ± 4.25	1.79 ± 1.51	−0.23	0.559
Hospital course				
In-hospital mortality, *n* (%)	19 (11.8)	12 (30.0)	0.46	0.0091
Mechanical ventilation, *n* (%)	53 (32.9)	17 (42.5)	0.20	0.273
Treatments				
Oseltamivir, *n* (%)	144 (89.4)	1 (2.5)	−3.57	<0.001
Ceftriaxone, *n* (%)	53 (32.9)	16 (40.0)	0.15	0.458
Azithromycin, *n* (%)	51 (31.7)	28 (70.0)	0.83	<0.001

Abbreviations: CRP, C-reactive protein; ICU, intensive care unit; IQR, interquartile range; RSV, respiratory syncytial virus; SD, standard deviation; SMD, standardized mean difference; SpO_2_, peripheral oxygen saturation. Values are expressed as mean ± SD, median (IQR), or *n* (%), as indicated. Analyses were based on available observations for variables with missing data; no imputation was used. Missingness affected heart rate (*n* = 1, 0.5%), respiratory rate (*n* = 4, 2.0%), SpO_2_ (*n* = 1, 0.5%), leukocyte count (*n* = 169, 84.1%), albumin (*n* = 67, 33.3%), and serum lactate (*n* = 40, 19.9%); CRP was complete. Continuous-variable SMDs were calculated from group means and the pooled standard deviation. For ICU length of stay and SpO_2_, SMDs were derived from the underlying means and standard deviations even though descriptive values are presented as median (IQR).

**Table 2:** Final multivariable logistic regression model for in-hospital mortality among ICU observations classified as RSV or influenza pneumonia. The model included infection type (RSV vs. influenza), age, sex, and serum lactate. Results are reported as adjusted odds ratios (ORs) with 95% confidence intervals (CIs). The analysis was based on 161 complete observations with 30 in-hospital deaths.

Variable	Adjusted OR	95% CI	*p*-Value
RSV pneumonia (vs. influenza)	6.03	2.08–17.49	0.001
Age (per year)	1.00	0.97–1.04	0.861
Male sex	0.24	0.08–0.70	0.009
Serum lactate (per mmol/L)	1.46	1.24–1.74	<0.001

Abbreviations: CI, confidence interval; OR, odds ratio; RSV, respiratory syncytial virus. Influenza pneumonia served as the reference category. The model was based on 161 complete observations with 30 in-hospital deaths.

**Table 3:** Stability of the association between RSV pneumonia and in-hospital mortality across regression models.

Model	*n*/Deaths	RSV OR (95% CI)	*p*-Value
Unadjusted	201/31	3.20 (1.40–7.34)	0.006
Adjusted for age and sex	201/31	3.13 (1.33–7.34)	0.009
Adjusted for age, sex, and lactate	161/30	6.03 (2.08–17.49)	0.001
Adjusted for age, sex, heart rate, and lactate	160/30	3.67 (1.17–11.53)	0.026
Firth logistic regression: age, sex, and lactate	161/30	5.59 (1.98–15.76)	0.001

Abbreviations: CI, confidence interval; OR, odds ratio; RSV, respiratory syncytial virus. Influenza pneumonia served as the reference category. Complete-case analysis was used for models including lactate. Heart rate refers to the earliest valid measurement after ICU admission.

**Table 4:** Performance indices of the final multivariable logistic regression model for in-hospital mortality among ICU observations classified as RSV or influenza pneumonia.

Performance Metric	Value
Area under the ROC curve (AUC)	0.864
Optimism-corrected AUC (bootstrap, 300 resamples)	0.845
McFadden’s pseudo-*R*^2^	0.285
Likelihood ratio test	<0.001

Abbreviations: AUC, area under the receiver operating characteristic curve; ROC, receiver operating characteristic. The table reports both the apparent AUC and the bootstrap optimism-corrected AUC based on 300 resamples.

**Table 5:** Sensitivity analyses of the association between RSV pneumonia and in-hospital mortality with additional adjustment for comorbidity burden and acute physiological severity.

Model	*n*/Deaths	RSV OR (95% CI)	*p*-Value
Adjusted for age and sex	201/31	3.13 (1.33–7.34)	0.009
Adjusted for age, sex, and Charlson Comorbidity Index	201/31	3.10 (1.32–7.29)	0.009
Adjusted for age, sex, Charlson Comorbidity Index, and ICD-based immunocompromised status	201/31	3.05 (1.30–7.15)	0.010
Adjusted for age, sex, heart rate, and serum lactate	160/30	3.67 (1.17–11.53)	0.026
Exclusion of overlapping RSV/influenza ICU admission; adjusted for age, sex, and lactate	159/30	6.34 (2.17–18.55)	<0.001

Abbreviations: CI, confidence interval; ICD, International Classification of Diseases; OR, odds ratio; RSV, respiratory syncytial virus. Influenza pneumonia served as the reference category. Heart rate refers to the earliest valid measurement after ICU admission. Models including serum lactate were restricted to observations with complete data for all covariates included in the respective model.

**Table 6:** Firth penalized logistic regression model for in-hospital mortality.

Predictor	OR	95% CI	*p*-Value
RSV pneumonia vs. influenza pneumonia	5.59	1.98–15.76	0.001
Age, per year	1.002	0.972–1.033	0.896
Male sex	0.267	0.096–0.743	0.011
Serum lactate, per 1 mmol/L increase	1.421	1.209–1.670	<0.001

Abbreviations: CI, confidence interval; OR, odds ratio; RSV, respiratory syncytial virus. Influenza pneumonia and female sex served as the reference categories. The model included 161 complete observations with 30 in-hospital deaths. *p*-values are based on Wald-type approximations for the Firth penalized estimates.

## Data Availability

The study data can be accessed through MIMIC-IV version 3.1 on PhysioNet (https://physionet.org/content/mimiciv/) after completion of the applicable credentialing and data-use requirements (accessed on 15 September 2025).

## Supplementary Materials

The following supporting information can be downloaded at: https://www.mdpi.com/article/10.3390/life16091533/s1, Supplementary Code S1, BigQuery SQL used to construct the MIMIC-IV analytical dataset; Supplementary Code S2, Python code for data cleaning, descriptive analyses, multivariable and Firth penalized logistic regression, sensitivity analyses, ROC analysis, and bootstrap internal validation; and a requirements file specifying the Python package versions used.
