# Supplementary material for: Clinical Outcomes of Respiratory Syncytial Virus Versus Influenza Pneumonia in Critically Ill Adults
Source: Life (Basel). 2026 Sep 15;16(9):1533. doi: 10.3390/life16091533 (PMC13608524; doi:10.3390/life16091533)
Supplement: Supplementary file 1 [file life-16-01533-s001.zip › life-4481824-supplementary.pdf]

REPRODUCIBILITY SUPPLEMENT

Clinical Outcomes of Respiratory Syncytial Virus versus Influenza Pneumonia in Critically Ill Adults

Contents

----

1. Supplementary_Code_S1_MIMICIV_BigQuery.sql
   BigQuery SQL for constructing an infection-specific ICU analysis dataset from MIMIC-IV v3.1.
2. Supplementary_Code_S2_Analysis.py
   Python analysis script for data cleaning, descriptive analyses, logistic regression,
   Firth penalized logistic regression, sequential/sensitivity analyses, ROC analysis,
   and 300-resample bootstrap optimism correction.
3. requirements.txt
   Exact Python package versions reported in the revised manuscript.

Data access

MIMIC-IV contains credentialed, de-identified clinical data and is not redistributed with this supplement. Users must obtain independent PhysioNet access and run the SQL against their authorized MIMIC-IV environment.

Analytic unit

The analysis uses infection-specific ICU observations. One ICU admission met criteria for both RSV and influenza and is represented once in each infection group in the primary cohort. The sensitivity analysis removes both infection-specific rows for that single overlapping ICU admission.

Case coding

The SQL contains the ICD-10 codes reported in the manuscript:

RSV: J12.1, B97.4

Influenza: J09.X1, J09.X2, J10.08, J10.1, J10.81, J11.08, J11.1

For the ICD-9 era, the query uses pneumonia-focused historical equivalents:

RSV: 480.1; RSV cause code 079.6 when accompanied by a pneumonia diagnosis.

Influenza with pneumonia: 487.0, 488.01, 488.11, 488.81.

Because the source manuscript evolved during revision, investigators should retain the executed SQL and exported cohort file with the final submission so that the exact database snapshot and code list remain auditable.

Expected principal results from the locked 201-observation analysis dataset

----

Unadjusted RSV OR: 3.20 (95% CI 1.40-7.34), p=0.006

Age/sex-adjusted RSV OR: 3.13 (95% CI 1.33-7.34), p=0.009

Age/sex/lactate-adjusted RSV OR: 6.03 (95% CI 2.08-17.49), p=0.001

Age/sex/heart-rate/lactate RSV OR: 3.67 (95% CI 1.17-11.53), p=0.026

Firth RSV OR: 5.59 (95% CI 1.98-15.76), p=0.001

Apparent AUC: 0.864

Optimism-corrected AUC, 300 bootstrap samples, seed 42: 0.845

Overlap-exclusion model: n=159, deaths=30; RSV OR 6.34

(95% CI 2.17-18.55), p<0.001

Charlson/immunocompromised sensitivity analyses

The SQL exports Charlson Comorbidity Index and an ICD-based immunocompromised flag. The Python script automatically runs these sensitivity models when those columns are present. These variables were not contained in the earlier locked reviewer spreadsheet; therefore their values must be regenerated from the authorized MIMIC-IV source using the SQL rather than inferred from the spreadsheet.

Run

BigQuery:

  Execute Supplementary_Code_S1_MIMICIV_BigQuery.sql after replacing

  YOUR_PROJECT.YOUR_DATASET with a writable destination.

Python:

  python Supplementary_Code_S2_Analysis.py --input rsv_influenza_icu_analysis.csv --outdir results

The script writes CSV files containing model estimates, missingness, model performance,

and Table-1-style descriptive summaries.

Software

Python 3.13.5

pandas 2.2.3

NumPy 2.3.5

SciPy 1.17.0

statsmodels 0.14.6

scikit-learn 1.8.0
